# Removal of *Staphylococcus aureus* from skin using a combination antibiofilm approach

**DOI:** 10.1038/s41522-018-0060-7

**Published:** 2018-08-06

**Authors:** Yi Wang, Xiaojuan Tan, Chuanwu Xi, K. Scott Phillips

**Affiliations:** 1 United States Food and Drug Administration, Office of Medical Products and Tobacco, Center for Devices and Radiological Health, Office of Science and Engineering Laboratories, Division of Biology, Chemistry and Materials Science, 10903 New Hampshire Ave, Silver Spring, MD 20993 USA; 2 Department of Environmental Health Sciences, School of Public Health, University of Michigan, 6631 SPH Tower, 1415 Washington Heights, Ann Arbor, MI 48109 USA

## Abstract

*Staphylococcus aureus* (*S. aureus*) including methicillin resistant *S. aureus* (MRSA) is one of the primary microorganisms responsible for surgical site infection (SSI). Since *S. aureus* contamination is known to originate from the skin, eradicating it on the skin surface at surgical sites is an important intervention to reduce the chance of SSIs. Here we developed and evaluated the efficacy of a combination probiotic/brush sonication strategy for skin preparation at surgical, injection and insertion sites in medicine. A 24 h biofilm on porcine skin explants was used as a worst-case scenario for the evaluation of preparation strategies. Conventional ethanol wipes achieved 0.8~2 log reduction in viable bacteria depending on how many times wiped (x4 or x6). Brush sonication or probiotic supernatant pre-treatment alone achieved a similar reduction as ethanol wipes (1.4 and 0.7~1.4 log reduction, respectively). Notably, combining sonication and probiotic pre-treatment achieved a 4 log reduction in viable bacteria. In addition, probiotic supernatant incubation times as short as 2 h achieved the full effect of this reduction in the combined strategy. These findings suggest the promising potential of combination-format skin preparation strategies that can be developed to more effectively penetrate cracks and folds in the skin to remove biofilms.

## Skin cleaning: sonication and probiotics can beat biofilms

Combining brush sonication with secretions from probiotic bacteria cleans skin before surgery more effectively than ethanol wipes. Researchers in the USA, led by K. Scott Phillips at the United States Food and Drug Administration, investigated removal of *Staphylococcus aureus* biofilm from pig skin as a “worst case” pre-surgical scenario. This bacterium is a major cause of serious and drug-resistant surgical site infections. Brush sonication or treatment with probiotic-derived solutions were individually approximately as effective as ethanol wipes, but in combination they proved substantially more effective. The treatment with the secretions surrounding probiotic bacterial cells requires exposure for several hours, but this could be readily achieved using a pre-surgery ointment. The sonication and probiotic combination could be developed into a highly effective pre-surgical procedure, penetrating cracks and folds in the skin to remove dangerous biofilms.

## Introduction

Surgical site infection (SSI) is the most common (160,000~300,000 per year) and most costly healthcare-associated infection^1^ in the United States and ranges from superficial skin infection to life-threatening postoperative complication. Foreign materials such as indwelling and implanted medical devices increase the risk of SSI significantly because less bioburden—as low as 100 CFU—is needed to cause infection.^2^ According to the 1999 CDC Guideline for Prevention of SSI, the endogenous microbes of a patient’s skin and mucous membrane are the primary source of pathogen contamination for most SSIs.^3^ Preventing initial bioburden transfer from the skin to foreign materials and adjacent tissue is thought to be an important intervention to prevent medical device associated SSI.^4,5^ However, current research on preventing medical device associated infections has focused more on antimicrobial biomaterials and sterile practices (such as handwashing) than on understanding how bioburden is transferred from the skin surrounding a penetration site. Therefore, understanding this aspect of the pathogenesis process can help inform the development of skin preparation countermeasures. By preventing contamination of normally sterile internal compartments, we can target the critical first step before bacterial colonization, multiplication and biofilm entrenchment. This could improve antimicrobial stewardship by reducing the use of antibiotics and antimicrobials.^6^

The human skin microbiota is diverse and includes numerous pathogenic bacteria.^7^ *Staphylococcus aureus* (*S. aureus*) are the most commonly isolated pathogen,^8^ accounting for 20–30% of SSI occurring in hospitals.^9^ This prevalence is related to the carriage of *S. aureus* in the healthy population (~20% persistent, ~60% intermittent).^10^ While topical antibiotics and antiseptics are often employed to reduce *S.aureus* colonization, these treatments may alter skin microbiota and reduce colonization by *S.aureus* competitors.^11^ Current patient-focused interventions to reduce contamination of surgical sites with pathogenic bioburden are limited to skin preparation and antibiotic prophylaxis. For surgical procedures at high risk of infection (contaminated wounds or dirty wounds), the use of prophylactic antibiotics has markedly reduced SSIs.^12^ However, the increasing spread of antibiotic resistant organisms makes prophylaxis more challenging and necessitates rethinking current approaches to improve stewardship of existing antibiotic resources. Considering that about 30% of infectious pathogens may be resistant to standard prophylactic antibiotics in the United States, as many as 120,000 SSIs and 6,300 deaths each year may be due to resistant organisms.^13^ The proportion of infections with resistant organisms is also on the increase.^14^

One way to reduce dependence on the use of antibiotics could be improved skin preparation to remove microbial counts to sub-pathogenic levels.^15^ Conventional skin preparation methods are widely accepted (alcohol, chlorhexidine, povidone-iodine and their combinations),^16,17^ and there are relatively few studies focused on improved approaches. Skin preparation strategies may benefit from other areas of infection control research, where an emerging approach to the treatment of biofilm involves the combination of physical forces—such as sonic energy or electric field--with antimicrobial treatment.^18–20^ These combined approaches are synergistic because the physical field helps break up biofilm structure while the antimicrobial component helps to kill segregated bacterial cells. In particular, the use of non-chemical antimicrobial approaches such as probiotics^21^ and phage^22^ is being explored to improve performance over conventional antimicrobials while benefiting antimicrobial stewardship. Since skin is colonized by endogenous bacteria, pretreatment with beneficial bacteria that already exist in healthy skin is potentially a safe and effective option.^23^ Beneficial probiotics compete with pathogens for adhesion and nutrients, weakening their ability to survive and proliferate. The supernatant produced by probiotic bacteria is rich in metabolites that are the likely source of antimicrobial activity against existing biofilms. For example, *Lactobacillus spp*. and *Bifidobacteria spp*. supernatants have been reported to reduce biofilm.^24,25^ Preliminary clinical studies have suggested that probiotic ingestion and nasal spray may be effective in eradicating persistent carriage of MRSA in the throat and nose.^26,27^ However, less has been studied about how probiotic strains might prevent biofilm associated infections, especially at surgical incision/injection sites.

In this work, we developed and assessed a combination approach for skin preparation. We used a recently developed porcine skin explant model^28^ to study the effectiveness of several alternative skin preparation approaches alone and in combination. The porcine skin model simulates a physiological tissue environment where pathogens may be more persistent than on abiotic materials.^29^ For example, *S. aureus* biofilm with host fibrin as part of the matrix have been shown to be more robust than on abiotic surfaces which are often used in in vitro testing.^30^ Since clinical testing is not possible with virulent pathogens, this approach provides a rapid, reproducible, and cost-effective way to test skin preparation strategies. We evaluated conventional alcohol-based skin wipes, a sonication brush, and probiotic bacteria, along with combinations of these approaches, for the potential to remove *S. aureus* growing in biofilm on the skin surface.

## Results

### Effect of alcohol wipe and brush sonication on skin *S. aureus* biofilm removal

**Fig. 1:**
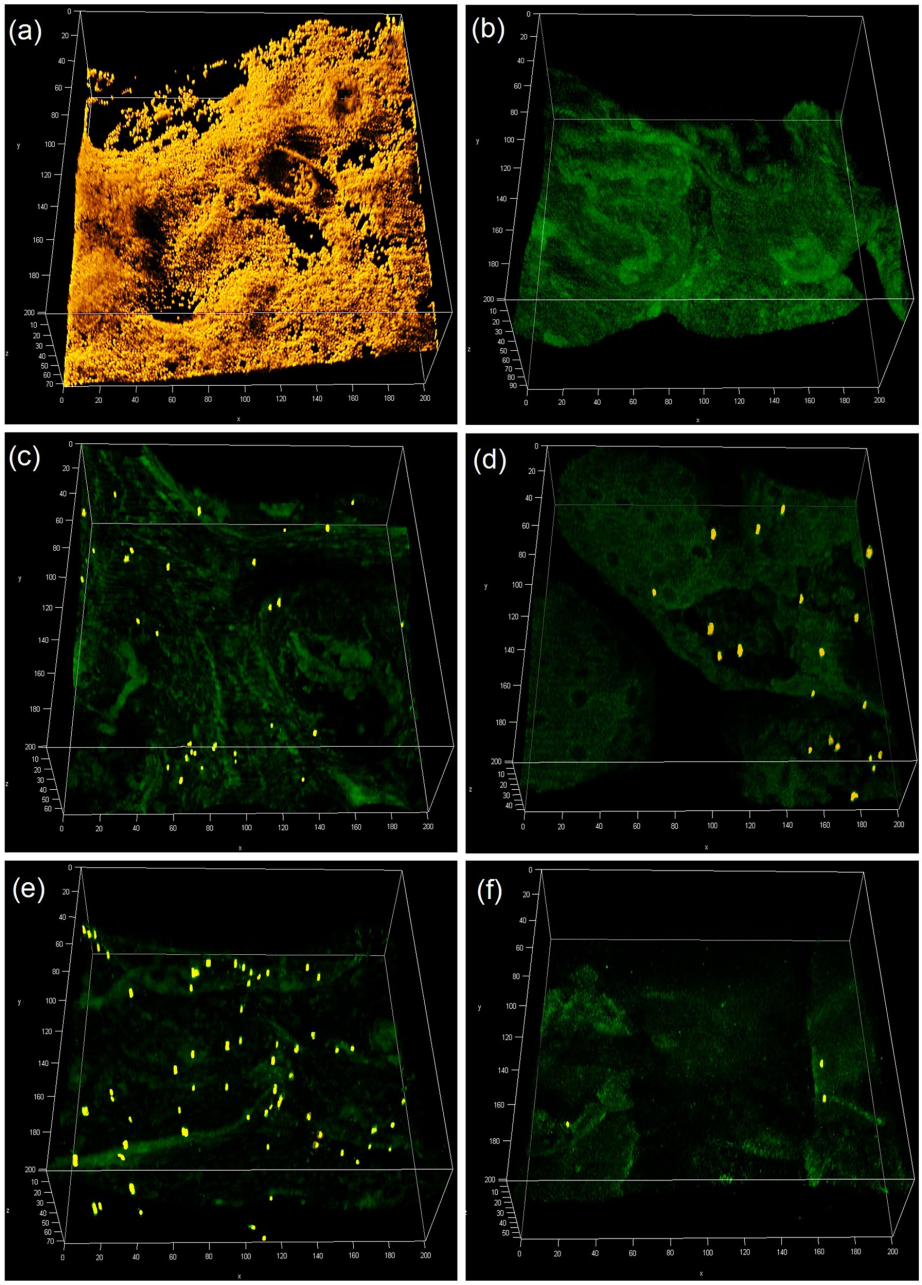
Confocal fluorescence image stacks of *S.aureus* biofilms on porcine skin before and after varied skin preparation treatments (200 µm x 200 µm x 60 µm) including 24 h biofilm as positive control **a**, porcine skin incubated with culture media as a negative control **b**, after alcohol wipes x4 **c**, brush sonication **d**, probiotic supernatant 2 h **e** and the combination treatment with probiotic & alcohol brush **f**. Scale bars are indicated in µm

**Fig. 2:**
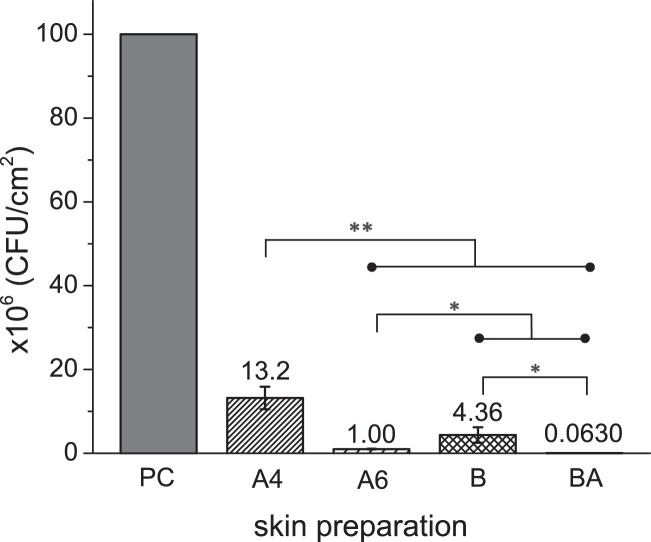
Number of viable *S. aureus* ( × 10^6^ CFU/cm^2^) on porcine skin surfaces before and after skin preparation with alcohol wipes (A4-alcohol wipes x4 and A6-alcohol wipes x 4), brush sonication (B) and combination treatment (BA- sonication brush with alcohol). Error bars represent the standard deviation over >3 different *S. aureus* cultures; *p < 0.05 and **p < 0.005. Raw data is available in Supplementary Materials, Data Availability Section

Porcine skin surface was inoculated with *S. aureus* (10^5^ CFU mL^−1^) and cultured 24 h for biofilm formation. The established bioburden was characterized with both CLSM imaging and plating. Before skin preparation (Fig. 1a), heterogeneous skin biofilm structure was observed. Both alcohol wipes (Fig. 1c) and brush sonication (Fig. 1d) were found to significantly reduce bioburden levels of *S. aureus* AH2547 24 h biofilm. To assess potential contamination from other microorganisms, plain porcine skin incubated with growth media was imaged after 24 h, and showed no bacterial growth (Fig. 1b). There were between 10^8^ to 10^10^ CFU cm^−2^ surface viable bacteria (PC) from different cultures and skin surfaces. After normalizing the viable number of PC to 10^6^ CFU cm^−2^ (Fig. 2), the 4 × alcohol wipe (A4), 6 × alcohol wipe (A6), sonication brush (B), and sonication brush with alcohol (BA) bioburden levels resulted in surface bacterial densities of (13.2 ± 2.7) × 10^6^, (1.00 ± 0.27) × 10^6^, (4.36 ± 1.8) × 10^6^, and (0.0630 ± 0.011) × 10^6^ CFU cm^−2^, respectively. A significant difference (*p* < 0.005) was seen when comparing A4 with A6, B, and BA. The three skin preparation methods (A6, B, and BA) were statistically different (*p* < 0.05), and BA removed 3 logs of bioburden.

### Effect of probiotic supernatant on skin *S. aureus* biofilm development and removal

**Fig. 3:**
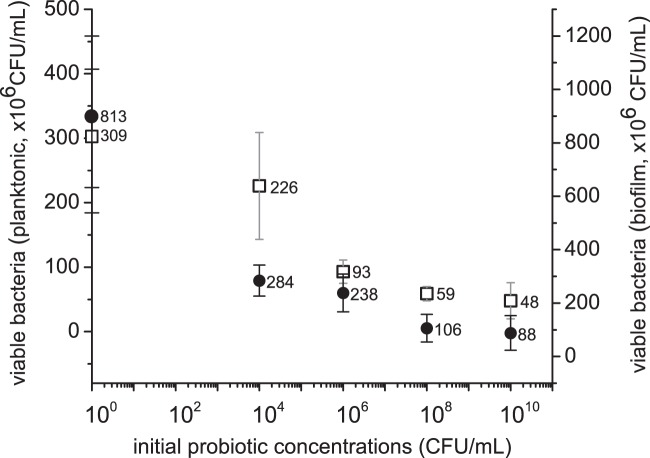
Probiotic supernatant *(Lactobacillus rhamnosus)* obtained from different initial cell densities (10^4^, 10^6^, 10^8^, 10^10^ CFU/mL) inhibits planktonic *S.aureus* (10^6^ CFU/mL) growth (□), and reduces 24 h *S. aureus* biofilm burden (●). Raw data is available in Supplementary Materials, Data Availability Section

LAB supernatant from multiple culture times was initially tested for inhibition of *S. aureus* growth to determine the optimal time (Supplementary Materials, Figure S1). Supernatants obtained at 16 and 24 h time points inhibited *S. aureus* growth for 24 h. We then tested how supernatants collected with different initial probiotic cell concentrations would inhibit *S. aureus* growth with the pig skin model (Fig. 3, hollow square). The results showed that inhibition of *S. aureus* growth increased (27, 70, 81, and 84%) with the starting inoculum of probiotic supernatants (10^4^, 10^6^, 10^8^, and 10^10^ CFU mL^−1^, respectively). The supernatants were also used to pre-treat well-established *S. aureus* biofilms (24 h) on skin (Fig. 3, solid circle). Compared to the control, supernatants collected from 10^4^ to 10^10^ CFU mL^−1^ *L. rhamnosus* inoculum reduced 65–89% of well-established skin surface bioburden.

### Combination treatment on skin *S. aureus* biofilm removal

**Fig. 4:**
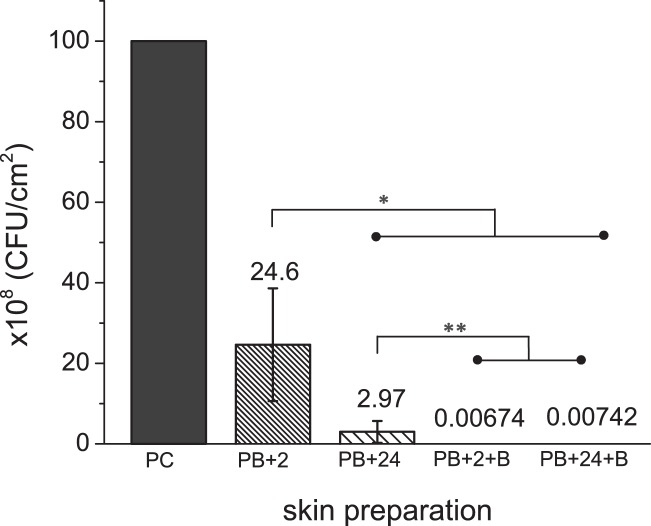
Number of viable *S.aureus* (×10^8^ CFU/cm^2^) on porcine skin surfaces before and after skin preparation with probiotic pre-treatment (2 and 24 h) and the combined treatments with brush sonication. Error bars represent the standard deviation over >3 *S.aureus* cultures; *p < 0.05 and **p < 0.005. The 24 h *S.aureus* biofilms were used as positive control, and the numbers after skin preparation were normalized to the positive control from the same batch of culture. Raw data is available in Supplementary Materials, Data Availability Section

Probiotic supernatant pretreatment of *S. aureus* biofilms for both 2 h (PB2 + 2) and 24 h (PB2 + 24) was evaluated (Fig. 4). After normalizing the viable number of PC to 10^8^ CFU cm^−2^, the PB2 + 2, PB2 + 24, and combined with brush sonication PB2 + 2 + B, PB2 + 24 + B bioburden levels resulted in surface bacterial densities of (24.6 ± 14) × 10^8^, (2.97 ± 2.7) × 10^8^, (0.00674 ± 0.0015) × 10^8^, and (0.00742 ± 0.0055) × 10^8^ CFU cm^−2^, respectively. With only probiotic pretreatment, the PB2 + 2 bioburden level is statistically different from the others (*p* < 0.05). While PB2 + 24 is significantly different from the other two combination treatments (*p* < 0.005), the difference in bioburden within the combination treatments (PB2 + 2 + B and PB2 + 24 + B) is not significant.

The bioburden removal efficacy (%) and log reduction for all tested skin preparation treatments on *S. aureus* 24 h biofilm were summarized in Table 1. Wiping more thoroughly with alcohol pads (A6 vs. A4) improved the removal efficacy from 86.8 to 98.9%, greater than one log increase. For skin preparation with the sonication brush, spraying the brush head with alcohol greatly helped biofilm removal compared to saline (~2 log improvement). Probiotic pretreatment alone reduced viable bacteria 75.4–97% (2–24 h). When combined with sonication brush/alcohol, the difference between probiotic pretreatment was minimized. Over 4 log reduction was achieved with combination treatment for well-established *S. aureus* biofilm (24 h) on porcine skin surfaces.

**Table 1:** Summary of the cleaning efficacy and log reduction for skin preparation treatments

	A4	A6	B	BA	PB2 + 2	PB2 + 24	PB2 + 2 + B	PB2 + 24 + B
Bioburden removal efficacy (%)	86.8 ± 2.7	98.9 ± 0.27	95.6 ± 1.8	99.9 ± 0.011	75.4 ± 14	97.0 ± 2.7	99.99 ± 0.0015	99.99 ± 0.0045
Viable bacteria log reduction	0.89 ± 0.09	2.01 ± 0.13	1.39 ± 0.20	3.23 ± 0.12	0.68 ± 0.31	1.38 ± 0.17	4.18 ± 0.10	4.27 ± 0.51

## Discussion

There is a need for better models to study how to improve skin preparation before penetrating procedures, to help reduce the chance of microbial contamination/infection in transcutaneous medical procedures. For infections associated with medical devices, the skin is a significant source of potential bioburden and could benefit from more effective preparation procedures. Due to our increasing understanding of the persistence of biofilm and its presence on human skin, it is important to test potential preparation strategies specifically against biofilm.

In this work, we used both plating and confocal microscopy (CLSM) to characterize bioburden after preparation of pig skin. Although CLSM is not ideal for quantifying large amounts of bacteria on the skin, it was valuable to show the distribution of bacteria after various cleaning procedures (Fig. 1). Unlike biofilms on smooth abiotic surfaces often used for in vitro effectiveness testing (plastic, silicone, etc.), biofilm on the rough topography of skin were heterogeneous and were made even more heterogeneous by the cleaning process. Bacteria remaining after alcohol wipes (3c) tend to be clustered in certain areas of the skin, likely at folds and ridges where the wipe was not able to make good contact. For the alternative skin preparation methods (3d–f) bacteria was left more homogenously scattered on the surface.

The roughness of skin and heterogeneous nature of cleaning are primary reasons why we tested sonication as an alternative to wiping as a physical removal method. Since increasing alcohol wipe steps from 4 × (A4) to 6 × (A6) significantly improved the reduction of bioburden (Fig. 2), we hypothesized that sonication might further help break up the biofilm matrix and work synergistically with other approaches to remove *S. aureus* contamination from skin. We know from previous results that there is little difference in skin wipes (alcohol, povidone-iodine and chlorhexidine) against *S. aureus* biofilm even among different types of antimicrobials.^28^ On the other hand, sonication is routinely used to remove bacteria from surfaces for enumeration, and biofilm removal products for teeth and skin based on sonication are considered clinically effective.^31,32^ In our experiments, sonication with saline alone was more effective than 4 × alcohol wipes. The oscillatory bristles with sonic energy were better at dislodging biofilms, which should translate into better real-world performance on skin folds and rough areas like wrinkles, facial pores and facial scars. The performance of sonication alone without alcohol as a confounding variable shows the importance of physical force in the cleaning process for biofilm in particular.

When alcohol was added to the sonication brush, removal of *S. aureus* was significantly better than even the 6 × alcohol wipe preparation. While sonication can physically remove biofilm, an antiseptic further reduces the bioburden through its biocidal effect on remaining microbes that are not easily removed. This explains why a more stringent wiping procedure with alcohol (i.e., 6×) was better than sonication alone. The importance of using an antiseptic has also been shown in studies of hand washing, where it was shown that 30-s rubbing with plain soap and water reduced *S.aureus* counts on contaminated hands by 29.9%, while application of 70% alcohol reduced *S. aureus* counts by 99.7%.^33^These results suggest that the heterogeneity of skin is indeed one of the challenges for conventional skin preparation methods, and show two possible ways to further improve this process, either by more stringent wiping or with a sonic delivery approach that is able to access crevices and break up biofilm.

While the use of more stringent physical preparation methods has promise to reduce existing skin bioburden of pathogenic bacteria, even 100 CFU/cm^2^ bioburden can cause contamination of dermal fillers when injected with certain styles or depths.^28^ As little as 500 CFU at a medical device implant site may be enough to cause infection.^34^ To further decrease the risks posed by skin contamination, we explored the addition of a probiotic step to the preparation process. Starting concentrations of a probiotic organism (*Lactobacillus rhamnosus* NBRC 3425) showed dose dependent effectiveness against planktonic and biofilm forms of *S. aureus* (Fig. 3). Supernatant obtained from overnight culture of an initial probiotic concentration of 10^4^ CFU/mL inhibit viable *S. aureus* in both planktonic and biofilm culture significantly (~30 and ~65%, respectively). While there was less reduction of the absolute amount of viable bacteria in the planktonic culture, this is likely because MRS culture media used for planktonic culture diluted the supernatant by half and also provided extra nutrient for bacterial growth. After increasing the initial probiotic concentrations used to produce the supernatant to 10^10^ CFU/mL, the viable *S.aureus* in planktonic and biofilm culture was further reduced by the treatment by 85 and 89%, respectively. This is likely due to the increased amount of exometabolites (bacteriocins, hydrogen peroxide, lactic acid, etc.), that exhibit both bactericidal and biofilm removal activities.^35,36^ The slope of the curve relating concentration to reduction in viable bacteria was similar for both forms of bacteria. While this reduction was only about 1 log, it was in the presence of a large initial inoculum of *S. aureus* over a short time period (24 h).

Next, the probiotic was tested in combination with the prior developed brush sonication strategy (Fig. 4). Since the time of exposure to the probiotic is important, two exposure times were evaluated (2 and 24 h). The 24 h time came from the idea that in principle a probiotic could be placed on the skin as an ointment the day before dermal filler injections would be performed, whereas the 2 h time point would be reasonable if the probiotic was placed on the skin the morning of a procedure. A worst case scenario (starting bioburden of 10^10^ CFU/cm^2^) was used for contamination. The impact of the probiotic for just 2 h was to reduce the bioburden by about 75%. At 24 h pre-treatment, the probiotic achieved nearly a 2-log reduction (3 × 10^8^ CFU/cm^2^), which was better than the reduction achieved with 4 × alcohol wipes and almost as good as that achieved with the sonication alone. When combined with sonication, the probiotic treatment showed an additive benefit. The reduction in bioburden was improved by an additional 3 orders of magnitude, for a total of almost 4.5 log reduction. Unexpectedly, the reduction in bioburden was similar for the 2 and 24 h treatment even though there was about 1 log difference with the probiotic treatment alone. Based on these results, it appears that the 2 h probiotic treatment combination with sonication brush was synergistic. In the presence of probiotic supernatant, biofilm structure may change within a couple of hours, which may make it easier to remove and kill *S. aureus* biofilm cells. Destruction of biofilm structure by the probiotic supernatant can be ascribed to various possible modes of action. One mode may be the presence of bacteriocin, antibiofilm metabolites, surfactants and some inhibitory *L. rhamnosus* compounds.^35–37^ These compounds disrupt the polymeric matrix of biofilm during formation, similar to other alternative strategies such as as the use of enzymes.^38^ Some studies have also suggested that the supernatant may contain organic acids that lower the pH, thereby inhibiting biofilm formation.^24^ In other studies the effect of pH on biofilm formation was not as pronounced, with other metabolites such as enzymes having a greater effect in particular on well-established communities.^39^ Regardless of the exact mechanism by which probiotics act against *S. aureus* in this case, it is important to emphasize that the use of an antiseptic such as alcohol is still essential after probiotic treatment because it has broad spectrum, tuberculocidal, fungicidal and veridical^40^ properties. The combination physical removal and probiotic intervention reported here is an additional method to reduce pathogenic bacterial burden and by itself does not have adequate broad spectrum properties to ensure safe skin penetration. Some probiotic strains may also become pathogenic inexplicably through unknown activation mechanisms and further study is needed to ensure that this or any potential probiotic therapy is safe before clinical use.

The tissue-based model employed here employs additional biological cues that are not found in current antimicrobial screening carried out in plastic microtiter plates. While the biofilm grown on porcine skin may not be identical to that found clinically, it represents a tradeoff necessary to achieve reproducible and controllable biofilms for quantitative assessment of skin preparation methods. Although the biofilms were only grown for 24 h, the inoculum challenge used (10^10^) greatly exceeds that typically found in the clinic. The combination method applied in this work has potential to be successful against real-world biofilms because beneficial bacteria such as *L. rhamnosus* are in theory already fit for competition with *S. aureus* and other microbes. In addition, since sonication is a mechanical force, it is not affected by whether a bacteria in biofilm is in a quiescent dormant state or fully active. It should have a similar impact on all biofilm with similar viscoelastic properties. For these reasons, we believe that our approach is more likely to be successful in a real-world application than the use of antibiotics, some of which we have found to be relatively ineffective against 24 h *S. aureus* biofilms even at multiples of the minimal inhibitory concentration. Although a final determination of effectiveness can only be obtained through a randomized controlled clinical trial, initial *in vivo* proof of concept might be obtained through minor modification of animal models developed for wounds.^41^

Currently, the effectiveness of skin preparation techniques using chemical approaches (antimicrobials) that don’t harm the skin is limited. The use of increased frequency or intensity of cleaning/washing^42,43^ has not shown significant improvements. The results of this study showed how a combination approach using a probiotic to help remove biofilm, combined with physical removal of bioburden can specifically address a persistent and high risk threat such as *S. aureus* growing in biofilm. This procedure could be combined with follow up antimicrobial wipes to address any remaining microbiological contaminants on the skin. While the procedure does add additional steps to the skin preparation process, it may be warranted in cases where reducing contamination to the lowest possible level is paramount, such as injections of dermal fillers, insertion of permanent implants or devices where contamination can result in colonization and biofilm formation leading to a persistent, drug resistant infection. This study fills a much needed gap in understanding the use of probiotics for skin preparation which was not addressed in current literature on “topical bacteriotherapy”, such as adding probiotics to skin care products to treat disturbed skin microbiota.^23^ Further work is needed to validate the benefit of this approach and develop standardized protocols that are clinically practical, safe and effective.

## Conclusion

Many surgical site infections caused by *S. aureus* initiate from bacterial transition from skin to the normally sterile internal tissues. Once biofilm is established, bacteria growing inside the matrix are highly resistant to antimicrobial agents. The emerging antibiotic resistance crisis compels us to reduce the incidence of these infections. Starting at the first step in pathogenesis, contamination, is logical because bacteria multiply exponentially and take shelter in biofilm, making them harder to eliminate over time. In this work, we developed a porcine skin biofilm model for testing skin preparation methods. Based on studies with this model, we suggest a combination of probiotic pre-treatment and brush sonication to both disrupt biofilm structure and physically remove *S. aureus* biofilm. This combination antibiofilm approach effectively reduces initial pathogenic bioburden and may help to maintain the balance of microflora on the skin when compared with the use of broad-spectrum antimicrobials. Combination strategies have been effectively used in other areas of infection control where a single treatment is insufficient, such as in treating antibiotic resistant infections,^44^ and against viral infections.^45^

## Materials and methods

### Porcine skin explant model

**Fig. 5:**
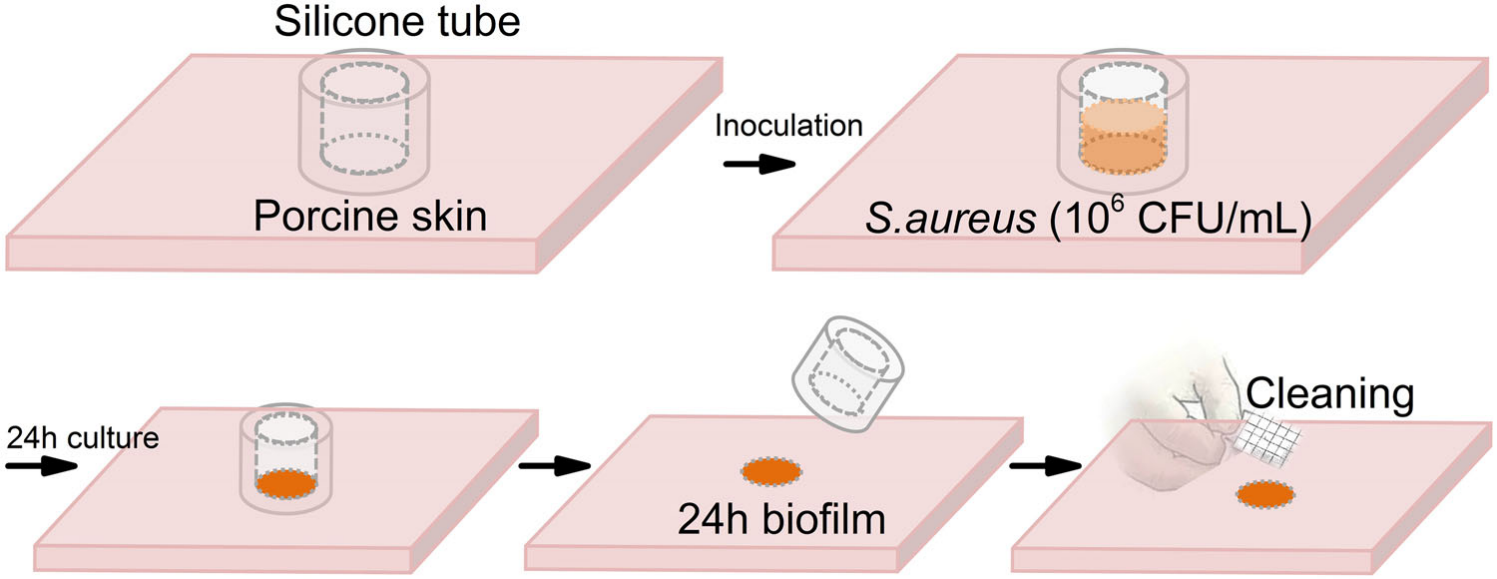
Biofilm model to assess skin preparation. Hand image adapted from Ref. ^28^. Image sourced from Wang Y., Leng V., Patel V., Phillips K.S., Scientific Reports 2017, published under the CC-BY license.

Porcine skin was prepared using established procedures (Fig. 5). Porcine skins ((Pel-freeze Biologicals, Rogers, AR) were grafted and cut to blocks with dimension of ~1″ x 1″ x 0.5″. Silicone tubing (autoclaved, 5/32″ID X 11/32″OD, Neoprene, Viton®, USA) was cut to 10 mm long and glued to skin blocks (one tube/ block). Neutral electrolyzed water (NEW) (Aquaox, Fontana, CA) was used to sterilize skin pieces. Diluted NEW solutions (HOCl, 80 mg/l) were applied twice (25 min/each) to the skin glued with tubing. The whole blocks were exposed to UV light in the biosafety hood for around 30 min to remove excess water on the surface. To test the biofilm removal efficacy, alcohol wipes, brush sonication, and probiotic pretreatment were applied on the 24 h biofilm formed with these substrates.

### Strains and biofilm culture conditions

The *Lactobacillus rhamnosus* (*L. rhamnosus)* used in this study was isolated from a commercial probiotic drink, by spreading 50 µl on MRS agar plates which were subsequently incubated at 37 °C for 48 h. After incubation, smooth convex whitish to creamy colonies were isolated and sub-cultured on Man-Rogosa-Sharpe (MRS; Difco) agar for 48 h. The strain was identified by 16 S rDNA sequencing. The 16 S rDNA sequence had 97% identity to *Lactobacillus rhamnosus* (*L. rhamnosus*) strain NBRC 3425 in the NCBI-database (National Center for Biotechnology Information), a lactic acid bacteria (LAB) that grows anaerobically but is aero-tolerant.^46^ LAB are present in healthy microbiota of the human gastrointestinal tract and are GRAS (generally recognized as safe).^47^ MRS broth (Difco) was used for culture and growth.

Green fluorescent protein tagged *Staphylococcus aureus* (*S. aureus*) AH2547 were provided by Dr. Alexander Horswill (Department of Biology, The University of Iowa, Iowa City, IA, USA).^48^ A colony of *S. aureus* was removed from the blood agar plate using an inoculating loop. The colony was placed in a centrifuge tube (15 mL) with 4 mL Tryptic Soy Broth (TSB) and vortexed for 10 s. The tube was incubated at 37 °C for 16~18 h with shaking (225 rpm). The culture was then vortexed and pushed through a 5μm filter to remove large clumps. The concentration was determined to be 10^8^ CFU/mL by plating on Luria-Bertani (LB) agar plates. The filtered culture was further diluted to ~10^5^ or 10^6^ CFU/mL in TSB and added to each tubing on the skin block. They were then incubated at 37 °C for 24 h. If not mentioned specifically, all media and agar plates used to culture *S. aureus* AH2547 had 10 µg/mL chloramphenicol to maintain the stability of plasmid^48^ and inhibit growth of native microorganisms on porcine skin.

### *S. aureus*-probiotic cell-free supernatant assay

Two assays were performed to assess the effects of probiotic supernatant on the inhibition of *S. aureus* growth and biofilm eradication, respectively. Both used cell-free supernatants obtained from *L. rhamnosus* overnight cultures filtered with a 0.22 µm membrane filter (EMD Millipore, Germany) after centrifugation. The initial inoculums were of 10^4^, 10^6^, 10^8^ and 10^10^ CFU/mL, to acquire controlled amount of inhibitory substances. The number of cells, ~10^10^ CFU/mL, in the overnight culture from one colony was determined by serial dilution and plating.

The *S. aureus* growth inhibition was then assessed by adding both 0.1 mL of *S. aureus* suspension (~10^6^ CFU/mL) and 0.1 mL of freshly prepared cell-free LAB supernatant to the tubing on porcine skin. The whole substrates were incubated for 24 h (37 °C). The number of viable *S. aureus* (planktonic), was acquired by scraping 5 times with 0.1 mL TSB. After serial dilution of the collected solution, 50 µL of each sample was spread on a 60 mm Luria broth (LB) agar plate with 10 µg/mL chloramphenicol and cultured for 24 h at 37 °C. In the positive control group, 0.1 mL of MRS broth was used instead of LAB supernatant.

The biofilm eradication efficacy was assessed by treating 24 h *S. aureus* biofilm with 0.2 mL LAB cell-free supernatant. The number of viable *S. aureus* cells (from biofilm) was acquired by plating as was described earlier. The positive control group contained 0.2 mL TSB and it was incubated with the other substrates for 24 h (37 °C).

### Skin preparation assays

**Fig. 6:**
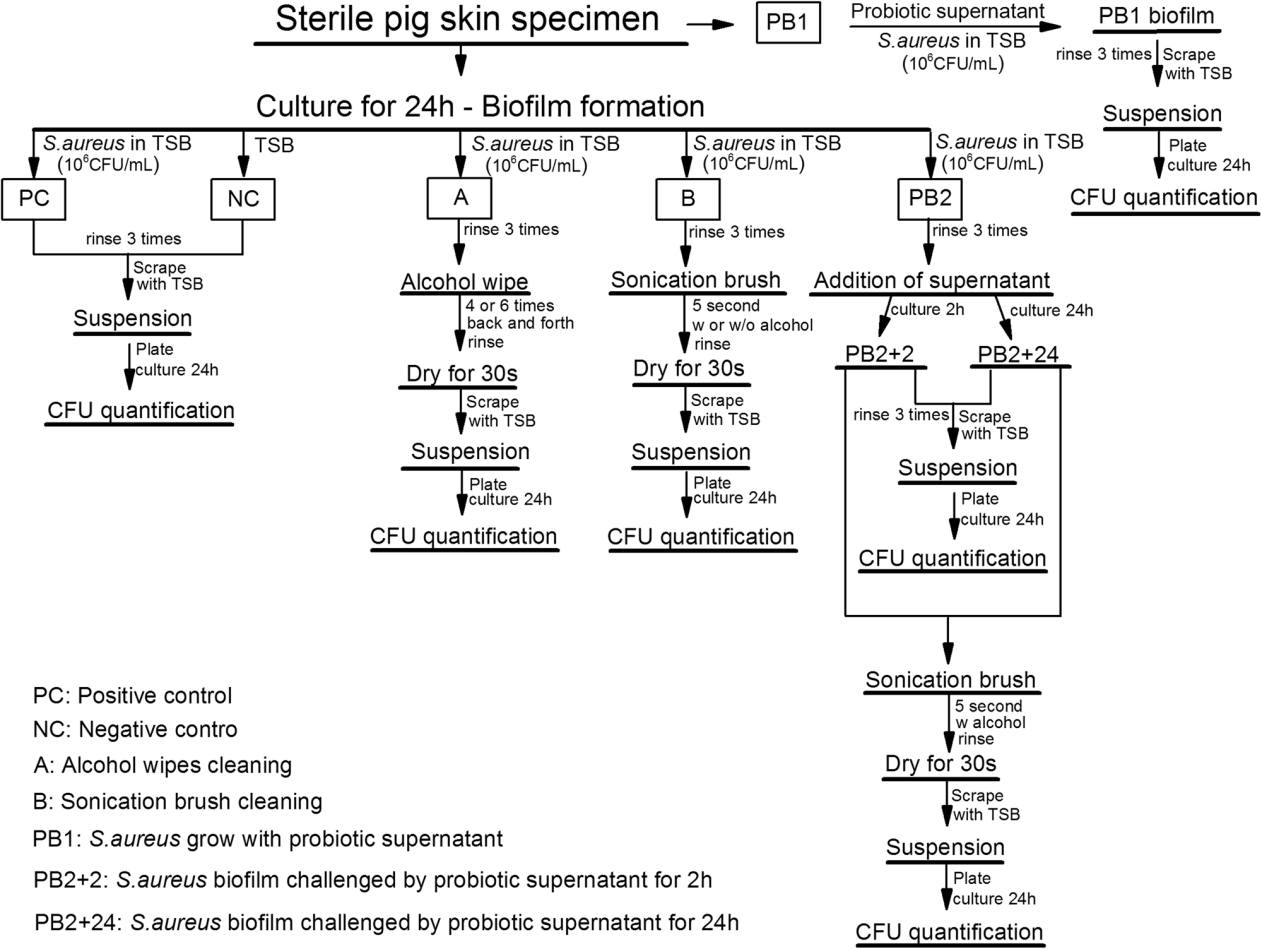
Flowchart of the skin preparation assays with experimental treatments, including alcohol wipe cleaning, sonication brush cleaning, alcohol sonication brush, probiotic supernatant pretreatment and combination (alcohol sonication brush with probiotic pretreatment)

Skin surfaces with *S. aureus* biofilm were obtained by inoculating exposed porcine skin surface in silicone tubing (0.2 mL of 10^6^ CFU/mL bacterial suspension) at 37 °C for 24 h (Fig. 6, positive control (PC)). Phosphate-buffered saline (PBS) was used to slightly rinse (3 times) the surface to remove loosely adhered cells. The attached surface cells were collected by scraping 5 times with 0.1 mL TSB/each. After serial dilution, 50 µL of each solution was spread on a 60 mm LB agar plate with 10 µg/mL chloramphenicol and then cultured for 24 h at 37 °C. Colonies of 20–200 for each plate were considered countable. Substrates cultured with 0.2 mL TSB with 10 µg/mL chloramphenicol were used as negative control.

The skin preparation with alcohol wipes (A) and brush sonication (B) were directly applied on the 24 h biofilm after rinsing. Wiping back and forth for 4 (A4) and 6 times (A6) were tested with alcohol wipes. For brush sonication, brush head sprayed with sterilized water (B) and 70% alcohol (BA) were compared. After treatment, skin substrates were dried for 30 s to remove excess surface water, and scraped with pipette tips. The collected bacterial solutions were plated and counted like the control groups.

The final combination therapy was carried out by applying sonication brush with alcohol spray on *S. aureus* biofilms after pre-treated with LAB supernatant for 2 h (PB2 + 2 + B) or 24 h (PB2 + 24 + B). The positive control group contained 0.2 mL TSB and it was incubated with the other substrates for 24 h (37 °C).

### Confocal laser scanning microscopy (CLSM) analysis

Imaging of bacterial biofilm on porcine skin surfaces before and after the treatment was performed with a Leica SP8 CLSM (Leica Microsystem, Germany). As illustrated in Fig. 6, porcine skin samples with biofilms before and after treatment were imaged directly after 3 × PBS rinse. CLSM images were collected with 485 nm excitation/535 nm emission. Simulated fluorescence projections through the biofilm were generated using the Leica LAS software.

### Statistical analysis

All assays were carried out at least three times with independently grown cultures unless otherwise stated. The results obtained were summarized in figures and tables as mean ± standard deviations. A one-way ANOVA was performed with post-hoc *t*-tests to determine significant differences. A *P*-value < 0.05 was considered significant.

### Data availability

All supporting data and datasets are available in the Supplementary Materials file.

## Electronic supplementary material

Supplementary Materials

## Supplementary information

**Supplementary information** accompanies the paper on the *npj Biofilms and Microbiomes* website (10.1038/s41522-018-0060-7).

## References

[1] Anderson DJ (2014). Strategies to prevent surgical site infections in acute care hospitals: 2014 update. Infect. Control. Hosp. Epidemiol..

[2] Moriarty TF, Grainger DW, Richards RG (2014). Challenges in linking preclinical anti-microbial research strategies with clinical outcomes for device-associated infections. Eur. Cell. Mater..

[3] Mangram AJ (1999). Guideline for prevention of surgical site infection, 1999. Hospital Infection Control Practices Advisory Committee. Infect. Control. Hosp. Epidemiol..

[4] Tsai DM, Caterson EJ (2014). Current preventive measures for health-care associated surgical site infections: a review. Patient Saf. Surg..

[5] Korol E (2013). A systematic review of risk factors associated with surgical site infections among surgical patients. PLoS One.

[6] O’Grady NP (2002). Guidelines for the prevention of intravascular catheter-related infections. Am. J. Infect. Control.

[7] Grice EA, Segre JA (2011). The skin microbiome. Nat. Rev. Microbiol..

[8] Jenks PJ (2014). Clinical and economic burden of surgical site infection (SSI) and predicted financial consequences of elimination of SSI from an English hospital. J. Hosp. Infect..

[9] Spagnolo AM (2013). Operating theatre quality and prevention of surgical site infections. J. Prev. Med. Hyg..

[10] Kluytmans J, van Belkum A, Verbrugh H (1997). Nasal carriage of *Staphylococcus aureus*: epidemiology, underlying mechanisms, and associated risks. Clin. Microbiol. Rev..

[11] SanMiguel AJ (2017). Topical antimicrobial treatments can elicit shifts to resident skin bacterial communities and reduce colonization by staphylococcus aureus competitors. Antimicrob. Agents Chemother..

[12] Finn Gottrup AM, Hollander DA (2005). An overview of surgical site infections: aetiology, incidence and risk factors. EWMA.

[13] Teillant A (2015). Potential burden of antibiotic resistance on surgery and cancer chemotherapy antibiotic prophylaxis in the USA: a literature review and modelling study. Lancet Infect. Dis..

[14] Weigelt JA (2010). Surgical site infections: Causative pathogens and associated outcomes. Am. J. Infect. Control.

[15] Greene LR (2012). Guide to the elimination of orthopedic surgery surgical site infections: an executive summary of the Association for Professionals in Infection Control and Epidemiology elimination guide. Am. J. Infect. Control.

[16] Maiwald M, Chan ES (2012). The forgotten role of alcohol: a systematic review and meta-analysis of the clinical efficacy and perceived role of chlorhexidine in skin antisepsis. PLoS One.

[17] Ngai IM (2015). Skin preparation for prevention of surgical site infection after cesarean delivery: a randomized controlled trial. Obstet. Gynecol..

[18] Qian Z, Stoodley P, Pitt WG (1996). Effect of low-intensity ultrasound upon biofilm structure from confocal scanning laser microscopy observation. Biomaterials.

[19] Ensing GT (2005). Effect of pulsed ultrasound in combination with gentamicin on bacterial viability in biofilms on bone cements in vivo. J. Appl. Microbiol..

[20] Khan SI (2016). Eradication of multidrug-resistant pseudomonas biofilm with pulsed electric fields. Biotechnol. Bioeng..

[21] Vesterlund S (2006). Staphylococcus aureus adheres to human intestinal mucus but can be displaced by certain lactic acid bacteria. Microbiology.

[22] Seth AK (2013). Bacteriophage therapy for Staphylococcus aureus biofilm-infected wounds: a new approach to chronic wound care. Plast. Reconstr. Surg..

[23] Nakatsuji T (2017). Antimicrobials from human skin commensal bacteria protect against Staphylococcus aureus and are deficient in atopic dermatitis. Sci. Transl. Med..

[24] Melo TA (2016). Inhibition of *Staphylococcus aureus* biofilm by *Lactobacillus* isolated from fine cocoa. BMC. Microbiol..

[25] Hor YY, Liong MT (2014). Use of extracellular extracts of lactic acid bacteria and bifidobacteria for the inhibition of dermatological pathogen *Staphylococcus aureus*. Dermatol. Sin..

[26] Roos K (2011). Can probiotic lactobacilli eradicate persistent carriage of meticillin-resistant *Staphylococcus aureus*?. J. Hosp. Infect..

[27] Gluck U, Gebbers JO (2003). Ingested probiotics reduce nasal colonization with pathogenic bacteria (Staphylococcus aureus, Streptococcus pneumoniae, and beta-hemolytic streptococci). Am. J. Clin. Nutr..

[28] Wang Y (2017). Injections through skin colonized with Staphylococcus aureus biofilm introduce contamination despite standard antimicrobial preparation procedures. Sci. Rep..

[29] Nemoto K (2000). Effect of varidase (streptokinase) on biofilm formed by *Staphylococcus aureus*. Chemotherapy.

[30] Kwiecinski J, Kahlmeter G, Jin T (2015). Biofilm formation by *Staphylococcus aureus* isolates from skin and soft tissue infections. Curr. Microbiol..

[31] Pitt WG (2005). Removal of oral biofilm by sonic phenomena. Am. J. Dent..

[32] Draelos, Z. D. Astringents, masks, and ancillary skin care products. in *Textbook of Cosmetic Dermatology* (eds Maibach, H. I. & Baran, R.) (CRC Press, 2010).

[33] Lilly HA, Lowbury EJL (1978). Transient skin flora—their removal by cleansing or disinfection in relation to their mode of deposition. J. Clin. Pathol..

[34] Poelstra KA (2000). Surgical irrigation with pooled human immunoglobulin G to reduce post-operative spinal implant infection. Tissue Eng..

[35] Petrova MI (2016). Lectin-like molecules of *Lactobacillus rhamnosus* GG inhibit pathogenic *Escherichia coli* and *Salmonella* biofilm formation. PLoS One.

[36] Rastogi P (2011). Probiotics and oral health. Natl. J. Maxillofac. Surg..

[37] Matsubara VH (2016). Probiotic lactobacilli inhibit early stages of Candida albicans biofilm development by reducing their growth, cell adhesion, and filamentation. Appl. Microbiol. Biotechnol..

[38] Romani AM (2008). Relevance of polymeric matrix enzymes during biofilm formation. Microb. Ecol..

[39] Jaffar N (2016). Mature biofilm degradation by potential probiotics: aggregatibacter actinomycetemcomitans versus *Lactobacillus spp*. PLoS One.

[40] William A. Rutala, D. J. W., and the Healthcare and I.C.P.A.C. (HICPAC). Guideline for disinfection and sterilization in healthcare facilities https://www.cdc.gov/infectioncontrol/guidelines/disinfection/index.html (2008).

[41] Jensen LK, Johansen ASB, Jensen HE (2017). Porcine models of biofilm infections with focus on pathomorphology. Front. Microbiol..

[42] Lilly HA, Lowbury EJ, Wilkins MD (1979). Limits to progressive reduction of resident skin bacteria by disinfection. J. Clin. Pathol..

[43] Larson EL, Eke PI, Laughon BE (1986). Efficacy of alcohol-based hand rinses under frequent-use conditions. Antimicrob. Agents Chemother..

[44] Lee CR (2013). Strategies to minimize antibiotic resistance. Int. J. Environ. Res. Public Health.

[45] Lebbink RJ (2017). combinational CRISPR/Cas9 gene-editing approach can halt HIV replication and prevent viral escape. Sci. Rep..

[46] Walstra, P., Wouters, J. T. M., Geurts, T. J. Dairy science and technology. in *Food Science and Technology* 2nd edn, (eds Walstra, P., Wouters, J. T. M., Geurts, T. J.) (CRC Press, 2005).

[47] Tripathi P (2012). Towards a nanoscale view of lactic acid bacteria. Micron.

[48] Pang YY (2010). agr-Dependent interactions of *Staphylococcus aureus* USA300 with human polymorphonuclear neutrophils. J. Innate Immun..

