# Supplementary material for: Removal of Staphylococcus aureus from skin using a combination antibiofilm approach
Source: NPJ Biofilms Microbiomes. 2018 Aug 6;4:16. doi: 10.1038/s41522-018-0060-7 (PMC6079078; doi:10.1038/s41522-018-0060-7)
Supplement: Supplementary file 1 — Supplementary Materials [file 41522_2018_60_MOESM1_ESM.pdf]

Removal of *Staphylococcus aureus* from skin using a combination antibiofilm approach (Supplementary Materials)

Yi Wang[1], Xiaojuan Tan[2], Chuanwu Xi[2], K. Scott Phillips[1]*

1.United States Food and Drug Administration, Office of Medical Products and Tobacco, Center for Devices and Radiological Health, Office of Science and Engineering Laboratories, Division of Biology, Chemistry and Materials Science, 10903 New Hampshire Ave, Silver Spring, MD 20993, USA

2. Department of Environmental Health Sciences, School of Public Health, University of Michigan, 6631 SPH Tower, 1415 Washington Heights, Ann Arbor, MI 48109, USA

*Corresponding author: kenneth.phillips@fda.hhs.gov

Supplementary Methods

Supernatant preparation *L. rhamnosus* supernatants obtained from one colony at different culture time (0, 2, 4, 8, 16, and 24h) were initially tested for inhibition of *S. aureus* (~10^6CFU/mL) growth in 96-well microtiter plates (supplementary test). Briefly, supernatants (0.1 mL/each) and *S. aureus* solution (0.1 mL, 10^6 CFU/mL) were added to each well and cultured for 24h at 37°C. Based on the result, we always use the probiotic supernatant collected after overnight (16-18h) culture if not mentioned specifically. We acquired the supernatants with a 0.22µm membrane filter (EMD Millipore, Germany) after centrifugation.

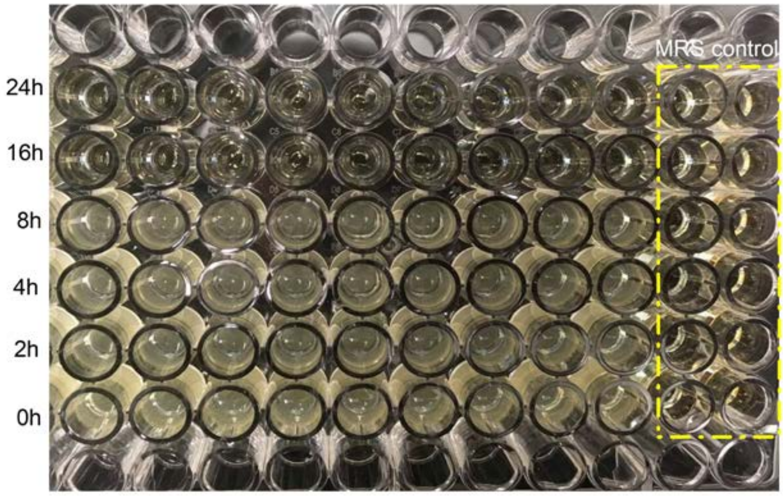

Supplementary Figure 1 (S1). Probiotic supernatant *(Lactobacillus rhamnosus* NBRC 3425*)* obtained from one colony and cultured for different time (0, 2, 4, 8, 16 and 24h) inhibit *S. aureus* (10^6 CFU/mL) growth. If not

mentioned specifically, all probiotic supernatant in subsequent intervention testing was obtained from overnight culture (16-18h).

Data Availability

Figure 4

PC	stdev	A4	stdev	A6	stdev	B	stdev	BA	stdev
100	0	13.2	2.69	1.00	0.268	4.36	1.82	0.0632	0.0107

Figure 5

initial probiotic concentrations	planktonic (10^6 CFU/mL)	stdev	biofilm (10^6 CFU/mL)	stdev
1.00E+10	48	28	88	65
1.00E+08	59	11	106	52
1.00E+06	93	18	238	71
1.00E+04	226	83	284	58
0	309	116	813	323

Figure 6

PC	stdev	PB2+2	stdev	PB2+24	stdev	PB2+2+B	stdev	PB2+24+B	stdev
100	0	24.6	14.0	2.97	2.73	0.00674	0.00154	0.00742	0.00546
